# Quantitative Trait Loci for Thermal Time to Flowering and Photoperiod Responsiveness Discovered in Summer Annual-Type *Brassica napus* L

**DOI:** 10.1371/journal.pone.0102611

**Published:** 2014-07-25

**Authors:** Matthew N. Nelson, Ravikesavan Rajasekaran, Alison Smith, Sheng Chen, Cameron P. Beeck, Kadambot H. M. Siddique, Wallace A. Cowling

**Affiliations:** 1 School of Plant Biology, The University of Western Australia, Crawley, Australia; 2 The UWA Institute of Agriculture, The University of Western Australia, Crawley, Australia; 3 Centre for Plant Breeding and Genetics, Tamil Nadu Agricultural University, Coimbatore, India; 4 National Institute for Applied Statistics Research Australia, University of Wollongong, Wollongong, Australia; University of Guelph, Canada

## Abstract

Time of flowering is a key adaptive trait in plants and is conditioned by the interaction of genes and environmental cues including length of photoperiod, ambient temperature and vernalisation. Here we investigated the photoperiod responsiveness of summer annual-types of *Brassica napus* (rapeseed, canola). A population of 131 doubled haploid lines derived from a cross between European and Australian parents was evaluated for days to flowering, thermal time to flowering (measured in degree-days) and the number of leaf nodes at flowering in a compact and efficient glasshouse-based experiment with replicated short and long day treatments. All three traits were under strong genetic control with heritability estimates ranging from 0.85–0.93. There was a very strong photoperiod effect with flowering in the population accelerated by 765 degree-days in the long day versus short day treatments. However, there was a strong genetic correlation of line effects (0.91) between the long and short day treatments and relatively low genotype x treatment interaction indicating that photoperiod had a similar effect across the population. Bivariate analysis of thermal time to flowering in short and long days revealed three main effect quantitative trait loci (QTLs) that accounted for 57.7% of the variation in the population and no significant interaction QTLs. These results provided insight into the contrasting adaptations of Australian and European varieties. Both parents responded to photoperiod and their alleles shifted the population to earlier flowering under long days. In addition, segregation of QTLs in the population caused wide transgressive segregation in thermal time to flowering. Potential candidate flowering time homologues located near QTLs were identified with the aid of the *Brassica rapa* reference genome sequence. We discuss how these results will help to guide the breeding of summer annual types of *B. napus* adapted to new and changing environments.

## Introduction

Timing of life history events (phenology) such as flowering time and maturity is crucial for the successful adaptation of a flowering plant to its environment [1]. The transition of the apical meristems from the vegetative to floral state involves a complex network of molecular signalling that integrates a range of environmental cues including daylength (photoperiod), prolonged cold associated with winter (vernalisation) and thermal responsiveness (also known as thermal time, thermal sensitivity, accumulated heat units or growing degree-days) [2]. Understanding how this complex process is mediated at the molecular level would provide useful tools for plant breeders to alter the adaptation of crops to new or changing environments.

The molecular control of floral initiation has been intensively studied in the model plant species, *Arabidopsis thaliana* (reviewed by [3]). Arabidopsis is a facultative long-day species and has both winter and summer annual types, and therefore serves as a useful model for understanding molecular control of flowering for many temperate crop species [4]. In Arabidopsis, key controller genes for vernalisation response are *FLC* and *FRIGIDA* and the key controller gene for photoperiod response is *CONSTANS* [3], [5]. Thermal responsiveness is not well characterised from a molecular standpoint but a recent reports implicated *PIF4*, *SVP* and *FLM* genes as key regulators of thermal response in Arabidopsis [5], [6], and a further five candidate genes were identified through association analysis in a panel of Arabidopsis accessions grown in different ambient temperatures [7]. Floral integrator genes – most notably *FT*, which encodes the mobile signal long-described as ‘florigen’ – then integrate these diverse signalling pathways to control flowering [8].

Rapeseed (*Brassica napus* L., also known oilseed rape or canola) is a close relative of Arabidopsis. Like Arabidopsis, rapeseed has both winter and summer annual types. Summer annual-type rapeseed has little or no requirement for vernalisation in order to flower, in contrast to winter annual-type rapeseed [9]. There are at least two sub-groups of summer annual rapeseed, one adapted to spring-sowing in Canada and northern Europe with warm, long days after sowing, and another adapted to autumn-sowing in southern Australia with cool, short days after sowing [9]. These two sub-groups have been reproductively isolated for many years and are genetically distinct [10], [11]. Summer annual-type rapeseed varieties are quantitative long day plants, which respond more or less to vernalisation and long days, but do not have an absolute requirement for either [12]. Flowering in Canadian summer-type rapeseed varieties was significantly delayed under short days [13], [14]. Australian summer annual rapeseed varieties showed a range of responsiveness to daylength and vernalisation but all varieties were very responsive to ambient temperature [15]. In that study, the variety Monty responded strongly to photoperiod and ambient temperature but weakly to vernalisation.

Phenological responsiveness to ambient temperature is observed across plant species and in animal species that cannot regulate their body temperature [16]. Between the baseline minimum and the optimum temperature, the relationship between development rate and temperature is normally linear. In *Brassica* species, the baseline temperature was calculated to be 0°C [15], [17] and so the accumulated thermal time to flowering can be simply calculated by adding the daily average temperature (in °C) for all the days up to the first day of flowering and expressed as degree-days. While the concept of thermal time to flowering is commonly employed in field-based agronomy and modelling studies, it has not been widely adopted by crop geneticists where time to flowering is normally expressed simply as the number of days to flowering. Therefore, in experiments carried out in non-constant temperature conditions (such as in the field or in basic greenhouse facilities) there is potential to enhance the characterisation of genetic factors underlying phenology by expressing development in thermal time units such as degree-days.

The genetic basis of flowering time control in *Brassica* species has been studied extensively by quantitative trait locus (QTL) analysis. Most studies have focused on populations with contrasting vernalisation responsiveness (e.g. [18], [19], [20], [21], [22], [23], [24]) with some exceptions (e.g. [25]). To our knowledge, no QTL analysis has been conducted on thermal time to flowering or photoperiod responsiveness in rapeseed, but such an approach proved to be effective in other crop species such as sunflower [26], rice [27] and sorghum [28]. The recent publication of the reference genome sequence for *B. rapa* [29] facilitates the association of candidate genes with flowering time QTLs in the *Brassica* A genome [20], [30]. The imminent availability of the genome sequences for *B. oleracea* and *B. napus* [31] will extend this capability to the C genome.

In this study, we investigated the genetic control of photoperiod responsiveness for flowering in a segregating doubled haploid population developed from a cross between homozygous lines derived from Australian and European summer annual rapeseed varieties. A glasshouse-based experiment was conducted in typical winter - spring growing conditions for southern Australia with and without supplemental lights to prolong daylength. Temperature measurements made throughout the experiment allowed accurate calculation of the thermal time to flowering for every plant in the experiment. Given the contrasting growing conditions experienced by summer annual rapeseed in Australia (grown over winter and spring) and Northern Europe (grown over spring and summer), and the reproductive isolation of these breeding pools, we expected that the Australian and European parents would contribute contrasting alleles for photoperiod sensitivity in flowering to the population. Using the published *B. rapa* genome sequence, we identified candidate flowering time gene homologues associated with several flowering time QTLs.

## Materials and Methods

### Plant material and glasshouse experimental design

The mapping population (LMDH) used in this study was a doubled haploid (DH) *B. napus* population (n = 131) derived from reciprocal F1 hybrids created from two genetically-distinct parents (European summer annual-type ‘Lynx-037DH’ and an Australian summer annual-type ‘Monty-028DH’ [32], [33]) using the method described by Cousin and Nelson [34]. The parents were homozygous DH lines developed by microspore culture from the varieties ‘Lynx’ and ‘Monty’, respectively. The F1-derived DH population was grown in a replicated glasshouse experiment along with the parents, reciprocal F1 hybrids and variety controls. The controls (kindly provided by Canola Breeders Western Australia Pty Ltd, Perth, Australia) included five summer annual-type varieties from diverse backgrounds: ‘Campino’ and ‘Topas’ (European varieties), ‘Westar-10DH’ (a DH line from Canadian variety ‘Westar’), ‘Telfer’ (a very early Australian DH variety), and ‘Tribune’ (a mid-season Australian DH variety).

**Figure 1:**
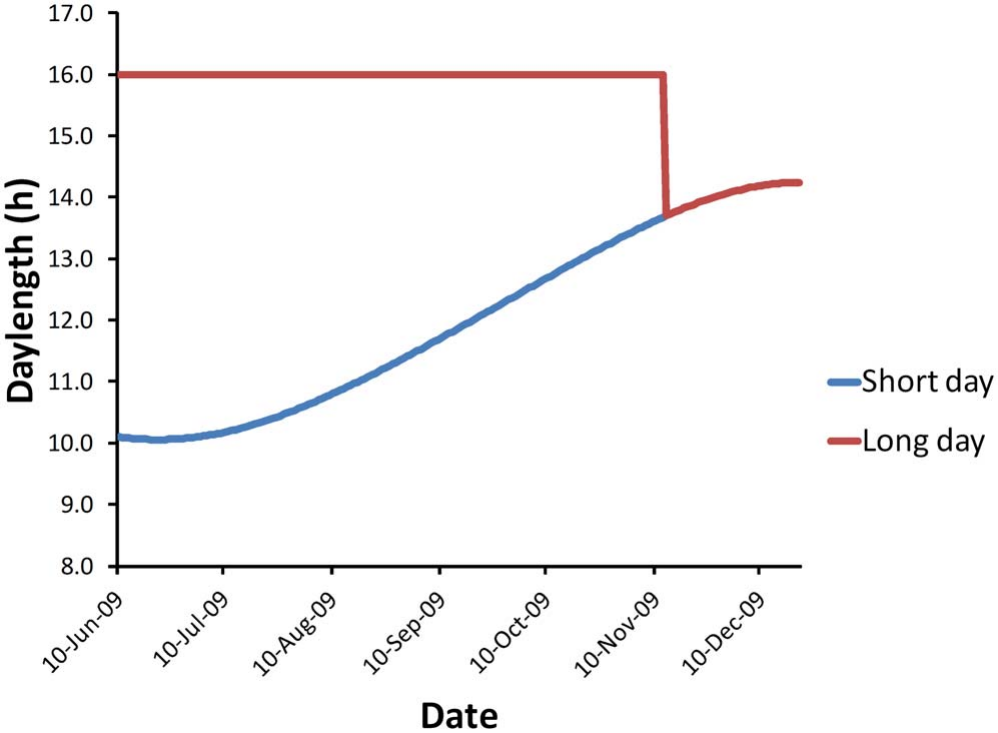
Daylength conditions for long day and short day treatments.

Seeds were sown on 10 June 2009 (approximately two weeks before the winter solstice) into potting mix in 50 mm×50 mm tall pots and placed on benches in a glasshouse at The University of Western Australia (Perth, Australia; latitude: 31°57′S; longitude: 115°52′E). Plants were watered daily throughout the experiment. There were four benches, each with 200 pots, arranged as 10 columns by 20 rows. The two day-length treatments were allocated to benches with two replicates (benches) per treatment. The entries were randomised to pots within benches using a partially replicated design [35] using DiGGeR [36] with the default pre-specified spatial model, allowing for random row and column effects. Parents and reciprocal F1s were duplicated on all benches, 35% of the LMDH population were duplicated in one out of four benches, 46% were duplicated in two out of four benches, and the remaining DH progeny and five control varieties were present as single entries on each bench (Table 1). In the short day (SD) treatment, plants received ambient daylight. Daylength at sowing was approximately 10 h and reached 12 h by day 101 of the experiment (Fig. 1; daylength data obtained from: http://www.timeanddate.com/worldclock/sunrise.html). Day time light intensity (photosynthetically active radiation, PAR; µmol.m^–2^.s^–1^) was recorded every 15 minutes and is summarised in Table 2. The two benches in the long day (LD) treatment had supplementary lights (two 12 W white fluorescent lamps per bench) in the evening to extend daylength to 16 h. Average light intensity under fluorescent lamps at the soil level was 1.94**±**0.53 µmol.m^–2^.s^–1^. Light pollution from the LD treatment benches, which may have affected neighbouring SD treatment benches, was prevented by drawing black curtains around the LD treatment benches at dusk every night. During the day, the curtains were drawn open to allow full sunlight on all LD and SD treatment plants. Supplementary lights in the LD treatment were withdrawn once all plants had flowered or were bolting with visible floral buds (13 November 2009, day 157 of the experiment). Ambient temperature in degrees Celsius was recorded at two points within the glasshouse every 15 minutes throughout the experiment. The change in season between winter (June to September) and spring (October to December) was reflected in the sharp temperature rise in the monthly mean temperature between September and October (Table 2). Days to germination, days to first flowering (DTF), thermal time to flowering (THERM) and the number of leaf nodes at first flowering (LNF) traits were recorded for all plants in the experiment. The average daily temperature for each 24 h period was calculated from the 15 minute temperature records, and the number of thermal units for each day of the experiment (expressed in degree-days) was calculated from the average daily temperature minus 0°C as the baseline temperature. The THERM trait score for each plant in the experiment was the number of accumulated thermal units on the first day of flowering of each plant, and was expressed in degree-days.

**Table 1:** Line and treatment replications used in this glasshouse experiment.

	Bench-1	Bench-2	Bench-3	Bench-4
Treatment^1^	SD-1	LD-1	LD-2	SD-2
Parents (n = 2)^2^	4	4	4	4
F1s (n = 2)	4	4	4	4
Controls (n = 5)	5	5	5	5
LMDH (n = 131)^3^	187	187	187	187
Total plants	200	200	200	200

1 Long day (LD) and short day (SD) treatments were duplicated.

2 Lynx-037DH and Monty-028DH parent were derived by microspore culture from Lynx and Monty varieties. Both parents were duplicated within each bench.

3 LMDH was a doubled haploid population generated from a cross between Lynx-037DH and Monty-28DH. LMDH lines were partially replicated within each bench (see text for details).

**Table 2:** Monthly temperature parameters (°C) and light intensity in this glasshouse-based flowering time experiment.

Month (2009)	Mean temp. (°C)	Mean day temp. (°C)	Mean night temp. (°C)	Hours below 12°C	Min. temp. (°C)	Max. temp. (°C)	Mean day PAR^1^ (µmol.m^–2^.s^–1^)
June	20.7	22.7	19.1	0.0	15.9	27.1	352
July	20.1	22.5	18.1	0.0	12.2	26.9	445
August	20.1	22.4	18.0	6.0	11.3	27.0	483
September	19.1	21.4	16.6	3.0	11.7	30.8	553
October	30.1	35.0	24.0	0.0	19.7	44.0	766
November	31.1	35.5	24.7	0.0	21.8	43.5	815
December	31.4	35.7	24.8	0.0	21.8	44.4	904

1 PAR = photosynthetically active radiation.

### Linkage mapping

The genetic map developed using this DH population was based on 135 SSR markers developed by Aslam [32], with an additional 437 DArT markers, six intron polymorphism markers and six gene-based markers including one flowering time gene marker, FLC3, in a genetic map for *B. napus* as reported by Raman et al. [33]. The total map length was 2,288 cM, which was estimated to encompass approximately 90% of the known *B. napus* genome. The linkage mapping strategy used to generate the linkage map [33] identified 329 non-redundant, high-quality framework markers in 19 linkage groups, which were used for QTL analysis in this experiment (Table S1).

### QTL analysis

The approach used in this study was a hybrid of those presented in Verbyla et al. [37], Pastina et al. [38] and Verbyla et al. [39] in which a linear mixed model analysis was employed to accommodate both genetic and non-genetic sources of variation. We commenced with the fitting of a baseline model (M0) in which the genetic effects for each treatment were partitioned into marker additive effects and polygenic effects (see Text S1 for full details). In brief, the marker effects related to the 329 marker positions and were fitted in the mixed model using the high dimensional approach of Stranden and Garrick [40]. The existence of the two treatments was commensurate with a bivariate structure so that for each set of genetic effects (that is, marker and polygenic) the model included a separate variance for each treatment and a covariance between treatments. Since a key aspect of this approach was the dual search for QTL main effects and QTL by treatment interactions, these bivariate parameters were also used to compute main effect and interaction variances. The non-genetic effects reflected the randomisation employed in the design and a spatial modelling component (of the form described in [41]) was included to allow for trends associated with the rows and columns within benches. The line effects for the DH population without marker data as well as parental lines and reciprocal F1s were fitted as fixed effects in order to exclude them from the sources of variation associated with the genetic effects.

After fitting the baseline model, denoted as M0, individual markers were scanned to establish a final multi-QTL model. The steps involved in this process were as follows:

1. In a manner similar to Pastina et al. [38], a sequence of 329 models was fitted in which M0 was modified by adding the marker main effect and marker by treatment interaction effect for the ith marker (i = 1.329) as fixed effects. It is important to note that by modifying M0 in this way, *all* marker and marker x treatment interaction effects are included simultaneously, and that effects for the ith marker are fitted as fixed effects, whereas the remainder are fitted as random effects. This differs from Pastina et al. [38] but is consistent with Verbyla et al. [37]. The model for the ith marker can be written schematically as M0+m_i_+m_i_×trt where m_i_ represents the (fixed) main effect of the marker and m_i_×trt the (fixed) marker by treatment interaction effect. For each model a *P*-value is obtained for the Wald statistic for both of these fixed effects. In order to respect marginality, the interaction effects were first examined. All marker by treatment interaction effects with *P*-values of less than 0.05 were chosen. The associated set of markers was then thinned to exclude markers with a high degree of collinearity using a threshold of 20 cM (Haldane; equivalent to 17 cM Kosambi). Suppose that the resultant set of markers is labelled a_1_…a_a_. The main effects and interactions for all markers in this set were then added to M0 as fixed effects to produce a working multi-QTL model, M1. Schematically this could be written as M1 = M0+a_1_+…+a_a_+a_1_×trt+…+a_a_×trt.
2. Step (1) was repeated but with M1 as the base model. Thus for each marker not already included with fixed effects in M1, the model M1+m_i_+m_i_×trt was fitted and significant marker by treatment interaction effects identified. Suppose that the resultant set of markers is labelled b_1_…b_b_. The sets of markers identified in (1) and (2) were combined and thinned for collinearity and the resultant set of main effects and interactions were fitted as fixed effects in working multi-QTL model M2. For ease of presentation of a schematic model, the case of no such collinearity is considered here, so that the model can be written as M2 = M0+a_1_+…+a_a_+b_1_+…+b_b_+a_1_×trt+…+a_a_×trt+b_1_×trt+…+b_b_×trt. This second scan was needed to ensure that the working multi-QTL model captured a substantial amount of marker by treatment interaction variance.
3. Steps (1) and (2) were repeated but in the context of examining marker main effects and commencing with M2 as the base model. This resulted in the identification of additional main effects to be added to the fixed effects to produce working multi-QTL model M3. Thus for markers c_1_…c_c_ identified in the first scan and d_1_…d_d_ in the second, and assuming no collinearity between them, the model is then given by M3 = M0+a_1_+…+a_a_+b_1_+…+b_b_+c_1_+…+c_c_+d_1_+… d_d_+a_1_×trt+…+a_a_×trt+b_1_×trt+…+b_b_×trt.
4. Backward elimination of fixed marker effects was then performed to obtain a parsimonious model. Once again, marker by treatment interaction effects were considered first, with backward elimination performed on M3. Effects were eliminated until the percentage of marker by treatment interaction variance fell to a nominated threshold. The resultant set of markers, that is, the subset of a_1_…a_a_, b_1_…b_b_ identified in this way, will be labelled as e_1_…e_e._ This produced working multi-QTL model M4 = M0+e_1_+…+e_e_+c_1_+…+c_c_+d_1_+…d_d_+e_1_×trt+…+e_e_×trt.
5. Step (4) was repeated on M4 for marker main effects. The resultant set of markers, that is, the subset of c_1_…c_c_, d_1_…d_d_ identified in this way, will be labelled as f_1_…f_f._ This produced the final multi-QTL model Mf = M0+e_1_+…+e_e_+f_1_+…+f_f_+e_1_×trt+…+e_e_×trt.
6. The final multi-QTL model was fitted to obtain Wald statistics for all marker by treatment interactions and main effects in the fixed part of the model. Effects that were significant at the *P* = 0.00001 level were deemed important and only these were reported. All other fixed marker effects in the final multi-QTL model were retained as ‘co-factors’ in the sense of composite mapping.

These analyses were conducted using ASReml [42].

### Alignment of genetic and physical maps for candidate gene identification

Alignment of the *B. napus* genetic map to the A-genome of *B. rapa* [29] was performed using BLASTn analysis (e-value<1e^−20^) of sequenced marker clones [43]. The locations of flowering time-related gene homologues in the *Brassica* A genome were determined by BLASTn analysis using coding sequences from 27 *Arabidopsis thaliana* genes listed in Table S2 (e-value<1e^−30^).

## Results

### Phenotypic analyses

**Figure 2:**
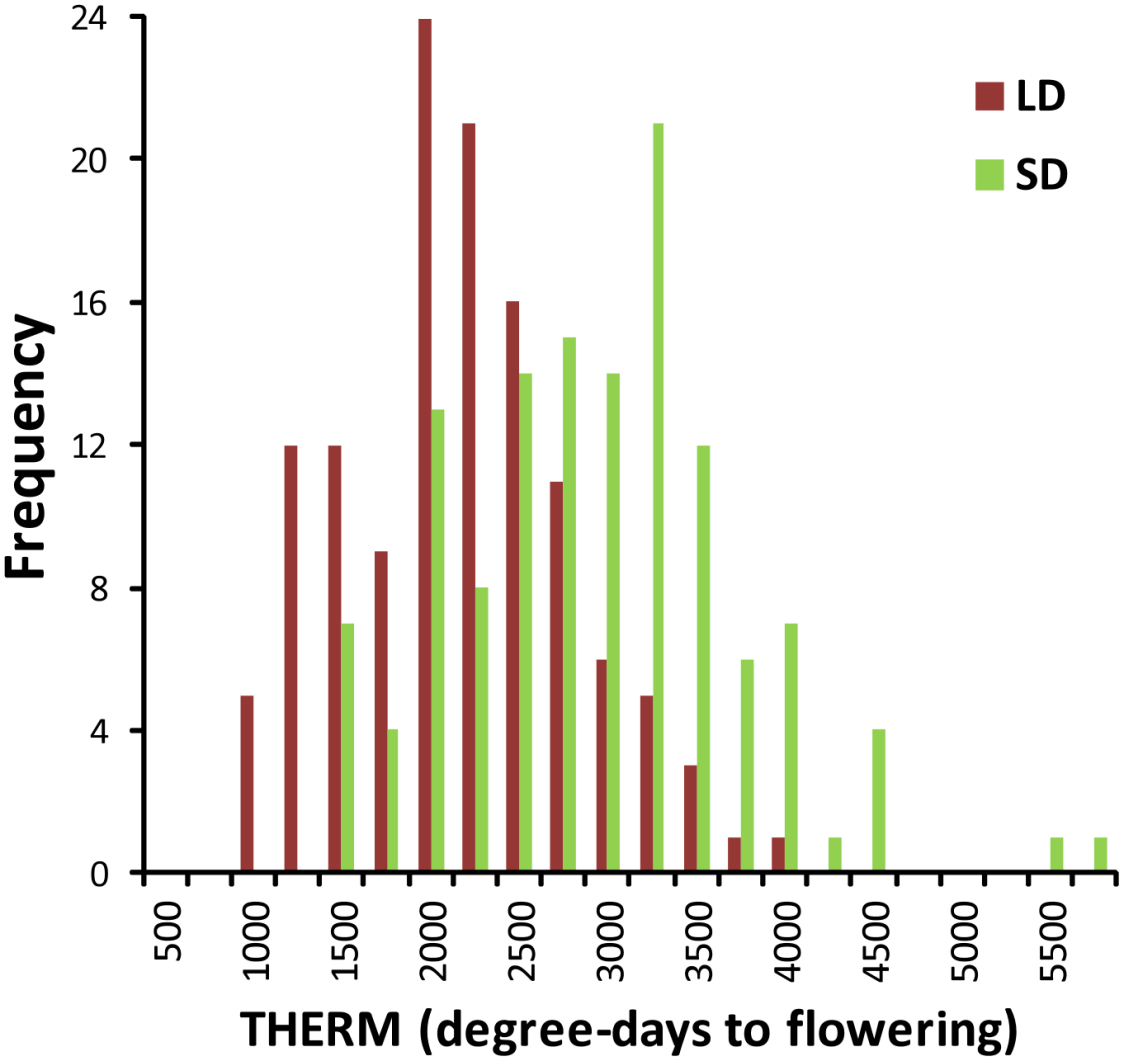
Frequency distribution for the thermal time to flowering (THERM) in the LMDH population. Plants were grown under long day (LD) and short day (SD) conditions in a glasshouse-based experiment. The mean THERM for Monty-028DH was 1839 degree-days (LD) and 2139 degree-days (SD). The mean THERM for Lynx-037DH was 2306 degree-days (LD) and 3094 degree-days (SD).

Seeds germinated successfully for all parents, F1 and control lines, and for 128 out of 131 DH lines, which were scored for thermal time to flowering (THERM), days to flowering (DTF) and leaf nodes at flowering (LNF) traits in long day (LD) and short day (SD) treatments (Table S3). A so-called phenotypic mixed model, that is model M0 without the inclusion of marker information, was used to determine appropriate spatial models and provide baseline treatment and genetic information for each trait. Plotting of residual errors for THERM, DTF and LNF showed that data for all traits in both LD and SD treatments were normally distributed (Figures S1–S3). All traits showed transgressive segregation in the Lynx-037DH x Monty-028DH doubled haploid (LMDH) population in both LD and SD treatments with the spread of predicted line means extending well beyond both parental means (Figs. 2, S4 and S5). The daylength treatment had a large significant effect (*P*<0.0001) for all traits; line means for all traits in LD and SD treatments are presented in Table 3.

**Table 3:** Line means and heritability estimates for flowering time-related traits in the LMDH population grown under long day (LD) and short day (SD) conditions.

	THERM^1^	DTF^2^	LNF^3^
LD mean	2029	97.2	14.8
SD mean	2794	128.0	22.6
*P*-value	<0.0001	<0.0001	<0.0001
LD heritability	0.91	0.91	0.85
SD heritability	0.92	0.93	0.89

1 THERM = thermal time to flowering (expressed as degree-days).

2 DTF = days to flowering.

3 LNF = leaf nodes at flowering.

The extent of genetic control of traits was then investigated by calculating line mean heritability for each treatment as the mean of the squared accuracy of the predicted DH line effects (see [44]). All traits were under strong genetic control in both LD and SD treatments with heritability ranging from 0.85 (LNF in the LD treatment) to 0.93 (DTF in the SD treatment) (Table 3).

The residual maximum likelihood (REML) estimates of the genetic variance parameters (that is, the LD and SD variances of line effects and the correlation between line effects from the two treatments) from the phenotypic mixed model for each trait are given in Table 4 along with the derived estimates of the line main effect variance and the line by treatment interaction variance. The percentages of total genetic variance explained by all markers for each trait and each source of variation were obtained by comparison with corresponding polygenic variance estimates from model M0. The main effect variance accounted for the major proportion of the variance, and markers explained a higher proportion of main effect variance (79.0 to 87.3%) than the interaction variance (57.6 to 60.6%) for the three traits THERM, DTF and LNF (Table 4). The line effects for all traits were highly correlated between LD and SD treatments ranging from 0.83 (LNF) to 0.91 (THERM), which was reflected in the predominance of main effect variance compared to interaction variance (Table 4).

**Table 4:** Genetic variance parameter estimates for flowering time-related traits from the phenotypic model (and % explained by all markers) in the LMDH population grown under long day (LD) and short day (SD) conditions.

Source of variation	THERM^1^	DTF^2^	LNF^3^
LD variance	446792 (92.5%)	837.2 (96.5%)	23.7 (84.8%)
SD variance	680869 (78.3%)	961.8 (80.4%)	58.9 (70.2%)
LD.SD correlation^4^	0.91	0.90	0.83
Main effect variance	499936 (87.3%)	806.8 (91.3%)	31.1 (79.0%)
Interaction variance	63895 (57.6%)	92.7 (58.3%)	10.3 (60.6%)

1 THERM = thermal time to flowering (expressed as degree-days).

2 DTF = days to flowering.

3 LNF = leaf nodes at flowering.

4 LS.SD correlation is the correlation of line effects grown under LD and SD conditions.

### QTL analyses

**Figure 3:**
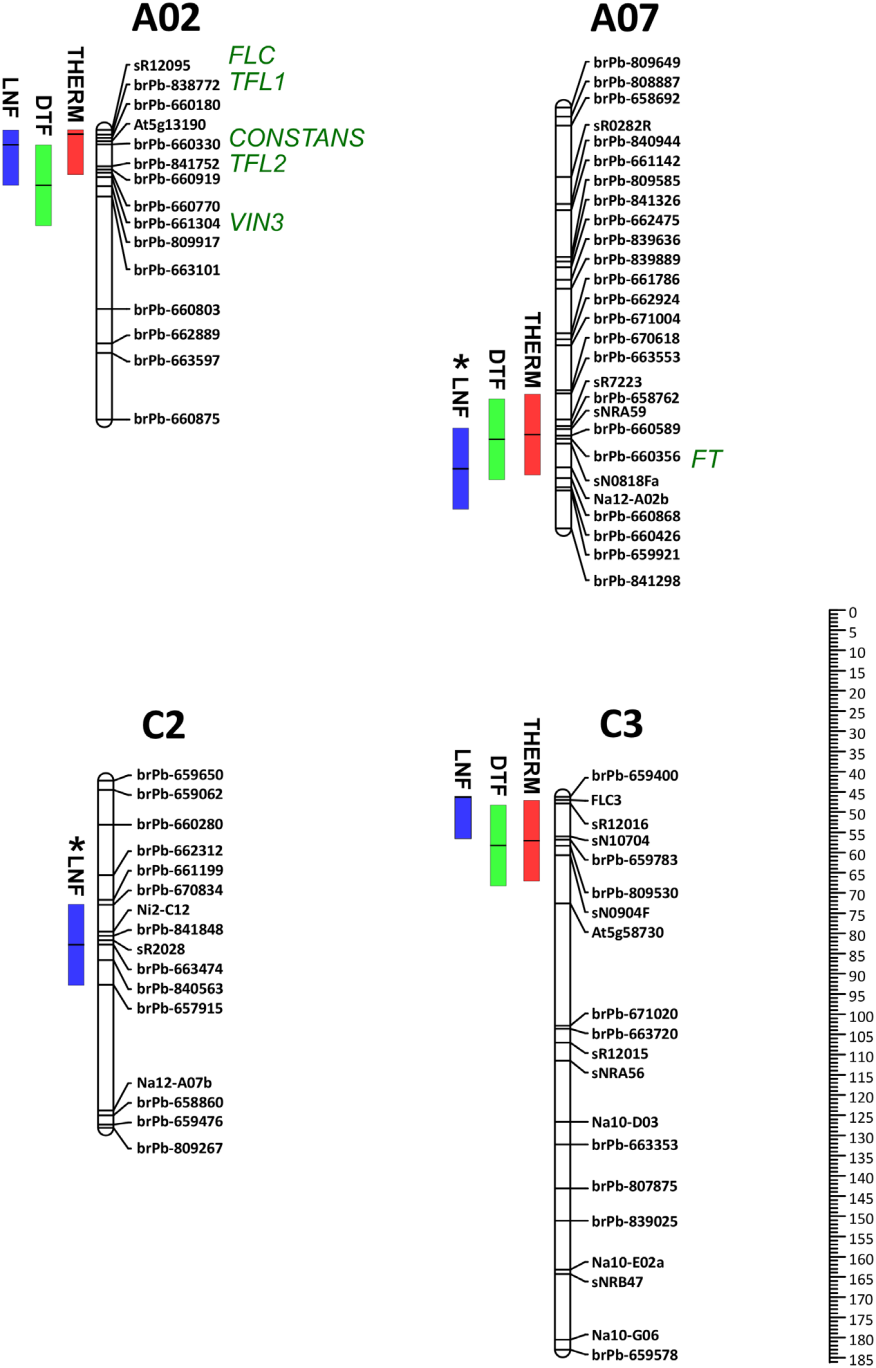
Distribution of flowering time-related QTLs in the LMDH framework map of *Brassica napus*. Flowering time traits comprised thermal time to flowering (THERM, expressed as degree-days), days to flowering (DTF) and number of leaf nodes at flowering (LNF). Linkage groups are drawn to Kosambi cM scale indicated in the scale bar. QTLs are represented as solid bars to the left of linkage groups with the central bar indicating the centre of each QTL and box showing 10 cM to each side of the centre. The predicted approximate locations of flowering time gene homologues near QTLs are shown to the right of the linkage groups.

The proportion of total variance that could be attributed to individual marker loci (i.e. QTLs) was then investigated for each trait. The probability of marker by treatment interactions (i.e. QTL×E) was first considered. The main effect was then calculated for those QTLs showing no interaction effect. Figure 3 summarises the distribution of QTLs on the LMDH genetic map.

#### Thermal time to flowering (THERM)

Three main effect QTLs controlling THERM were detected which together accounted for 57.7% of variance in both LD and SD treatments (Table 5). Both parents contributed alleles for earlier flowering and random segregation of allelic effects at these three QTL indicates a potential range in THERM of 1041 degree-days above and below the mean in the population.

**Table 5:** Summary of significant (*P*<0.00001) quantitative trait locus positions and effects (QTLxE interactions and main effects) for flowering time traits measured in the LMDH population grown under long day (LD) and short day (SD) conditions.

Trait	Marker	Chr.	Pos. (cM)	*P* (QTLxE)	*P* (main effect)	LD effect^4^ (PEV^5^)	SD effect (PEV)	Main effect (PEV)
THERM^1^	brPb-838772	A02	1.2	ns^6^	0	-	-	+433 (29.1%)
	brPb-660589	A07	81.2	ns	6.11e-09	-	-	–296 (13.5%)
	brPb-809530	C3	12.1	ns	1.94e-12	-	-	–312 (15.1%)
	**NET effect (total PEV)**				**-**	**-**		**–175 (57.7%)**
	**Range** 7					**-**	**-**	**±1041**
DTF^2^	brPb-809917	A02	13.9	ns	2.22e-16	-	-	+17.0 (26.6%)
	brPb-660356	A07	82.0	ns	1.92e-06	-	-	–11.2 (11.6%)
	brPb-809530	C3	12.1	ns	4.24e-11	-	-	–12.5 (14.5%)
	**NET effect (total PEV)**				**-**	**-**		**–6.7 (52.7%)**
	**Range**					**-**	**-**	**±40.8**
LNF^3^	brPb-660330	A02	3.6	ns	0	-	-	+3.8 (39.2%)
	Na12-A02b	A07	89.1	3.73e-06	-	0 (0%)	–3.1 (15.7%)	
	brPb-663474	C2	40.6	5.17e-07	-	0 (0%)	+2.6 (10.9%)	-
	brPb-659400	C3	0.0	ns	8.32e-11	-	-	–1.9 (10.0%)
	**Net effect (total PEV)**				**0 (0%)**	**–0.5 (26.6%)**		**+1.9 (49.3%)**
	**Range**					**-**	**±5.7**	**±5.7**

1 THERM = thermal time to flowering (expressed as degree-days).

2 DTF = days to flowering.

3 LNF = leaf nodes at flowering.

4 Positive effect values indicate that the European (Lynx-037DH) allele delayed flowering while negative effect values indicate that the Australian (Monty-028DH) allele delayed flowering.

5 PEV = percent explained variance.

6 ns = not significant (*P*>0.00001).

7 Range indicates the potential range above and below the means of the respective traits conditioned by the presented QTLs.

#### Days to flowering (DTF)

Three main effect QTLs controlling DTF were detected which together accounted for 52.7% of variance in both LD and SD treatments (Table 5). All three DTF QTLs were located close to the THERM QTLs (Fig. 3). Both parents contributed alleles for earlier flowering and random segregation of allelic effects at these three QTL indicates a potential range in DTF of 40.8 days above and below the mean in the population.

#### Leaf nodes at flowering (LNF)

Four QTLs controlling LNF were detected which together accounted for 63.3% of the variance in the LD treatment and 90.4% in the SD treatment (Table 5). Three LNF QTLs were located close to THERM QTLs (Fig. 3). There were two main effect QTLs where the net effect of alleles derived from Monty-028DH was to reduce LNF by 1.9 in both treatments. Two interaction effect (i.e. photoperiod responsive) QTLs were detected where the net contribution of Monty-028DH alleles increased LNF by 0.5 in the SD treatment. Both parents contributed alleles for earlier flowering and random segregation of allelic effects at these three QTL indicates a potential range in LNF of 5.7 above and below the respective means in the population in long days and 11.4 in short days.

### Candidate genes for flowering time QTLs

Linkage groups A01–A10 of the *B. napus* LMDH genetic map were aligned to the *B. rapa* A-genome sequence via BLASTn analysis of sequenced genetic markers (Table S4). The physical location of flowering time homologues was similarly determined using Arabidopsis gene coding regions as the query sequences (Table S2). On the basis of co-location of markers and flowering time homologues in the *B. rapa* genome potential candidate genes underlying QTLs could be identified for all 6 QTLs that mapped to A-genome chromosomes (Fig. 3). There was a cluster of flowering time homologues in the vicinity of THERM, DTF and LNF QTLs on chromosome A02, notably *FLC*, *CONSTANS*, *TFL1*, *TFL2* and *VIN3*. The region of A07 containing QTLs for THERM, DTF and LNF was near the predicted location of an *FT* homologue. In addition, the *FLC* homologue-based molecular marker FLC3 mapped to the same location as the cluster of THERM, DTF and LNF QTLs on C3.

## Discussion

This is the first report of thermal time to flowering (THERM) QTLs in summer annual-types of rapeseed. Despite the very compact nature of the experiment, spatial analysis improved accuracy of the estimation of line effects, and heritability estimates of THERM were extremely high: 0.91 in long days (LD) and 0.92 in short days (SD) (Table 3). The strong correlation between line effects in LD and SD (0.91, Table 4) strengthened the determination of main effect QTLs across the LD and SD treatments through bivariate analysis.

Photoperiod had a profound effect on flowering, which was accelerated by an average of 765 degree-days in LD compared with SD treatments (Table 3). However, there was a high correlation between line effects in LD and SD, the main effect variance (LD vs SD treatment) was much greater than interaction variance, and markers explained more of the main effect than interaction variance (Table 4). From these results we concluded that both parents shared similar photoperiod responsiveness alleles. This was unexpected, given the contrasting environments to which summer annual rapeseed is adapted in Australia (winter-sowing) and in Europe (spring-sowing). Our data support previous photothermal modelling of flowering time in Australian-type summer annual type rapeseed varieties which found high photoperiod responsiveness in Monty and other Australian varieties [15]. To our knowledge, equivalent information is unavailable for European summer annual rapeseed varieties, other than Lynx, so that inferences from this study to other European varieties should be made with caution.

Given the genetic distinctiveness of Australian and European summer annual type rapeseed varieties [10], [11], some segregation of flowering times was expected in the LMDH. However, the extreme extent of transgressive segregation for THERM and other flowering time traits was surprising (Figs. 2, S4 and S5). Much of that variation was conditioned by three main effect QTLs explaining 57.7% of the variation in the THERM trait, and allelic effects at these QTL accounted for a potential range in THERM of 1041 degree-days above and below the mean in the population, with both parents contributing alleles for accelerated and delayed flowering (Table 5). The THERM main effect QTLs helped to explain the large transgressive segregation observed in the DH population in LD and SD treatments (Fig. 2), and the high genetic correlations between the two treatments (Table 4).

It is unclear at this stage which floral initiation pathway accounted for the non-photoperiod related (i.e. main effect) QTLs. While both Australian and European rapeseed parents used in this experiment were summer annual types, residual vernalisation requirement could conceivably underlie some of these QTLs. The minimum temperature in this glasshouse-based experiment was 11.3°C and there were just 9 hours below 12°C in the whole growing period, which would be insufficient to fulfil vernalisation requirements. However, the variety Monty (from which the Australian parent Monty-028DH was derived by microspore culture) is known to have a very low vernalisation requirement but is highly responsive to changes in ambient temperature [15]. Further experiments are planned using controlled temperature experiments both with and without vernalisation pre-treatments to separate the potentially confounding effects of responsiveness to vernalisation and ambient temperature. Any remaining genetic variation not captured by these environmental cue-related QTL could arise by other endogenous factors that operate independently of environmental cues.

In this study, we included two other measures of flowering time: days to flowering (DTF), which is the normal measure of flowering time in previous rapeseed flowering time studies; and the number of leaves produced by each plant prior to flowering (leaf nodes at flowering, LNF), which is more commonly used in the Arabidopsis research community [5]. Both measures had very high heritability estimates in the LMDH population in both LD and SD treatments (>0.85; Table 3). There was considerable congruence in the locations of THERM, DTF and LNF QTLs where three sets of QTLs for all three traits clustered together on chromosomes A02, A07 and C3. There was one further LNF QTL on chromosome C2 (Fig. 3), which highlights the usefulness of multiple measures of flowering time in order to capture more of the genetic variation for flowering time in rapeseed. Interestingly, two of the LNF QTLs showed significant interaction effect (Table 5) although this result should be treated with caution given the relatively low overall interaction variance compared to main effect variance (Table 4).

The availability of a reference sequence for the *Brassica* A-genome allowed the identification of flowering time homologues for all 6 QTLs located on A-genome chromosomes, and an *FLC*-based molecular marker co-localised with three QTLs in the C-genome (Fig. 3). The three strongest effect QTLs for all three flowering time-related traits were associated with key regulator genes. The floral integrator gene *FT* was located near the THERM/DTF/LNF QTLs on chromosome A07. Functionally, *FT* is a plausible candidate given its role is the integration of multiple flowering pathways [8]. Homologues of the floral repressor *FLC* mapped to the same location as two major QTLs on chromosomes A02 and C3. *FLC* is most commonly associated with vernalisation response in Arabidopsis and rapeseed [3], [45]. However, the recent report by Xiao et al. [30] may point to a broader regulatory role for *FLC* in *Brassica* species and so could conceivably be involved in thermal responsiveness. It should be noted that there were several other flowering time homologues near the QTLs on A02, and the imminent release of a reference sequence for the *Brassica* C genome [31] may uncover other flowering time homologues in the QTL region on C3. Further linkage and association QTL analyses are essential to more precisely define the positions of flowering time QTLs in order to find more robust associations with candidate genes. Functional and variant characterisation of candidate genes could then be used to gauge the likelihood that these genes are indeed responsible for the major effects on flowering time in rapeseed.

Most previous *Brassica* flowering time QTL studies have been conducted using crosses between summer and winter-types where vernalisation response typically dominates variation in flowering time (e.g. [18], [21], [23], [46], [47]). The *FLC*-associated QTLs on linkage groups A02 and C3 in this study (Fig. 3) map to similar genetic locations as vernalisation-associated QTLs previously identified in *B. rapa* [46], [47], *B. oleracea* [23] and *B. napus* [19], [20]. Interestingly, the similarly-positioned QTL on A02 observed by Raman et al. [20]) was observed only when that winter annual rapeseed population had been vernalised. It is unclear if these collective results support the conclusion of Xiao et al. [30] of multiple roles for *FLC* given the presence of other flowering time homologues in the vicinity of *FLC* (Fig. 3; Table S2). However, it is clear that we need to move beyond imprecise mapping of QTLs to the identification of causal genes underlying QTLs if we are to directly compare genetic control of flowering time across populations and across *Brassica* species.

Genetic variability in photoperiod and thermal responsiveness to flowering will become increasingly important to plant breeders as they develop varieties suited to a warming global climate and expansion of rapeseed cultivation into higher or lower latitudes. The extreme transgressive segregation of flowering time traits observed in the LMDH population (Figs. 2, S4 and S5) demonstrates the wealth of flowering time diversity available to rapeseed breeders if they are prepared to cross between the previously-isolated Australian and European breeding pools [11]. If the variation detected in the LMDH population is representative of the broader European and Australian breeding pools, rapeseed breeders who wish to develop spring-sown varieties for higher latitude regions of Europe could access the very wide genetic diversity in flowering time that is generated by segregation among several QTLs in crosses between European and Australian varieties. Conversely, breeders could develop varieties for longer-season autumn-sowing regions of Australia by accessing late-flowering alleles from European varieties. Further studies using a wide range of European and Australian summer-annual rapeseed varieties under controlled environment conditions will be required to confirm the general applicability of our findings and could potentially identify additional sources of variation for responsiveness to environmental cues including photoperiod. The QTLs identified in this study, together with diagnostic molecular markers, will equip breeders with the tools required to adapt summer annual rapeseed to new and changing climates.

## Supporting Information

Figure S1 **Histogram of residual errors for thermal time to flowering (THERM) in the LMDH population.** Plants were grown under long day (LD) and short day (SD) conditions in a glasshouse-based experiment.
(TIF)

Figure S2 **Histogram of residual errors for days to flowering (DTF) in the LMDH population.** Plants were grown under long day (LD) and short day (SD) conditions in a glasshouse-based experiment.
(TIF)

Figure S3 **Histogram of residual errors for the number of leaf nodes at flowering (LNF).** Plants from the LMDH population were grown under long day (LD) and short day (SD) conditions in a glasshouse-based experiment.
(TIF)

Figure S4 **Frequency distribution for days to flowering (DTF) in the LMDH population.** Plants were grown under long day (LD) and short day (SD) conditions in a glasshouse-based experiment. The mean DTF for Monty-028DH was 90.5 days (LD) and 104.4 days (SD). The mean DTF for Lynx-037DH was 110.6 days (LD) and 141.0 days (SD).
(TIF)

Figure S5 **Frequency distribution for number of leaf nodes at flowering (LNF) in the LMDH population.** Plants were grown under long day (LD) and short day (SD) conditions in a glasshouse-based experiment. The mean LNF for Monty-028DH was 15.3 (LD) and 18.8 (SD). The mean LNF for Lynx-037DH was 18.4 (LD) and 23.9 (SD).
(TIF)

Table S1 **Graphical genotype information for 128 lines in the** ***Brassica napus*** **LMDH population.** Data are presented for 329 non-redundant, high quality framework markers on 19 chromosomes (A01–A10 and C1–C9).
(XLSX)

Table S2 ***Arabidopsis thaliana*** **flowering time gene homologues and BLASTn matches in the** ***Brassica rapa*** **genome.**
(XLSX)

Table S3 **Raw data for flowering time-related traits in the LMDH population and controls.** Flowering time traits comprised thermal time to flowering (THERM, expressed as degree-days), days to flowering (DTF) and number of leaf nodes at flowering (LNF).
(XLSX)

Table S4 ***Brassica napus*** **LMDH linkage groups A01–A10 aligned to the** ***Brassica rapa*** **genome.**
(XLSX)

Text S1 **Base-line marker model, M0, in asreml syntax.**
(DOCX)
