# Supplementary figures and images for: Quantitative Trait Loci for Thermal Time to Flowering and Photoperiod Responsiveness Discovered in Summer Annual-Type Brassica napus L
Source: PLoS One. 2014 Jul 25;9(7):e102611. doi: 10.1371/journal.pone.0102611 (PMC4111298; doi:10.1371/journal.pone.0102611)

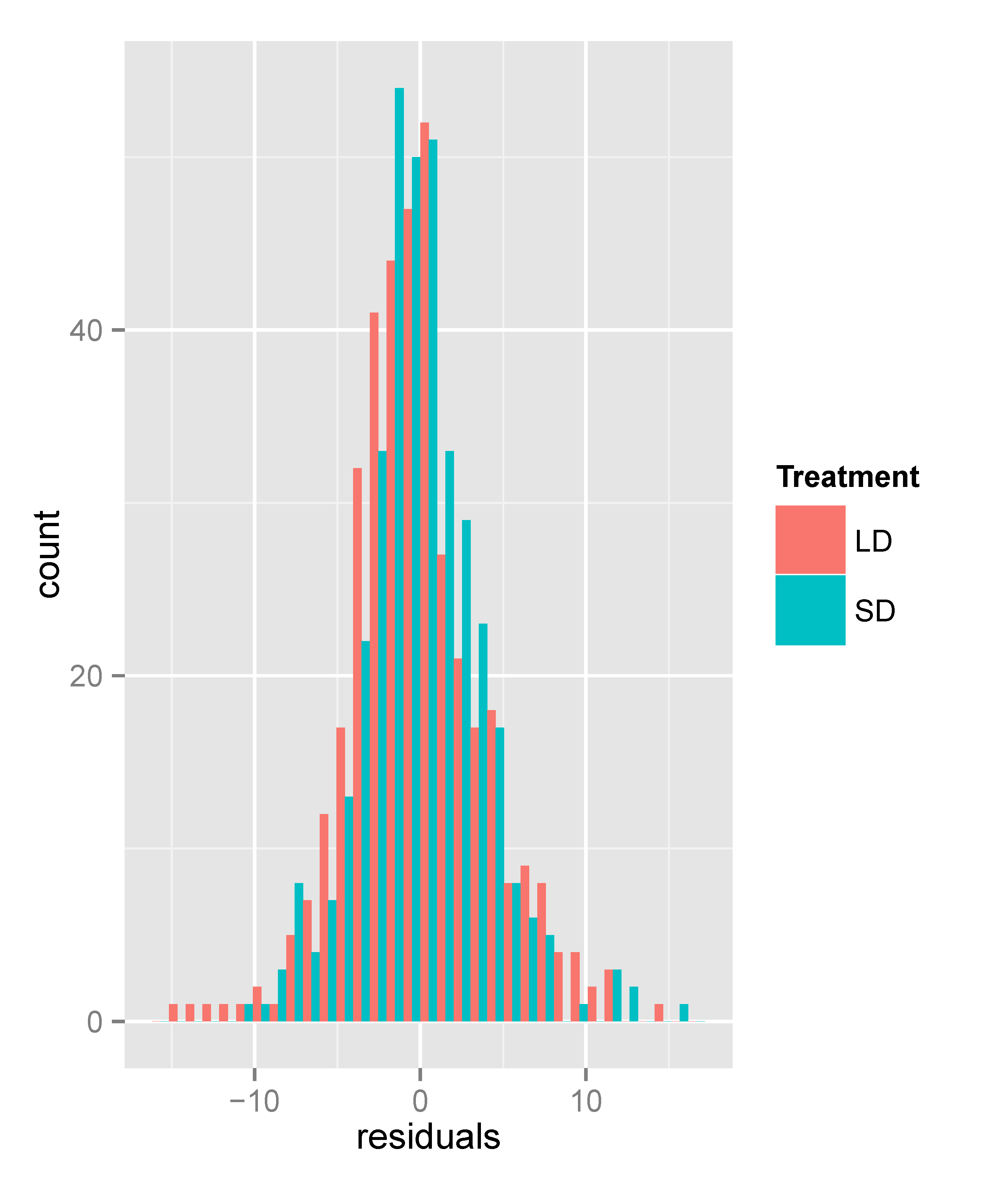

Supplement: Figure S1 — Histogram of residual errors for thermal time to flowering (THERM) in the LMDH population. Plants were grown under long day (LD) and short day (SD) conditions in a glasshouse-based experiment. (TIF) [file pone.0102611.s001.tif]

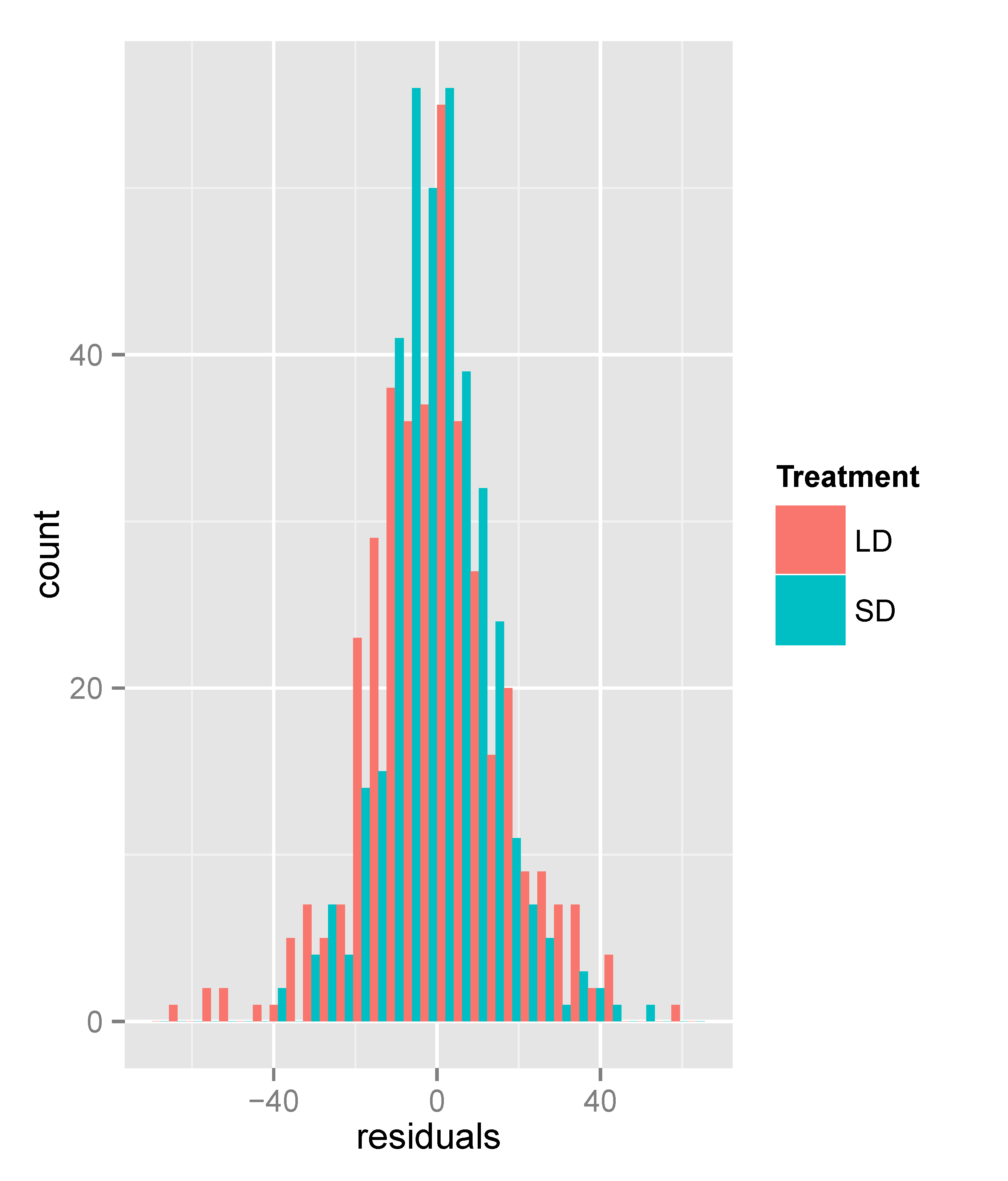

Supplement: Figure S2 — Histogram of residual errors for days to flowering (DTF) in the LMDH population. Plants were grown under long day (LD) and short day (SD) conditions in a glasshouse-based experiment. (TIF) [file pone.0102611.s002.tif]

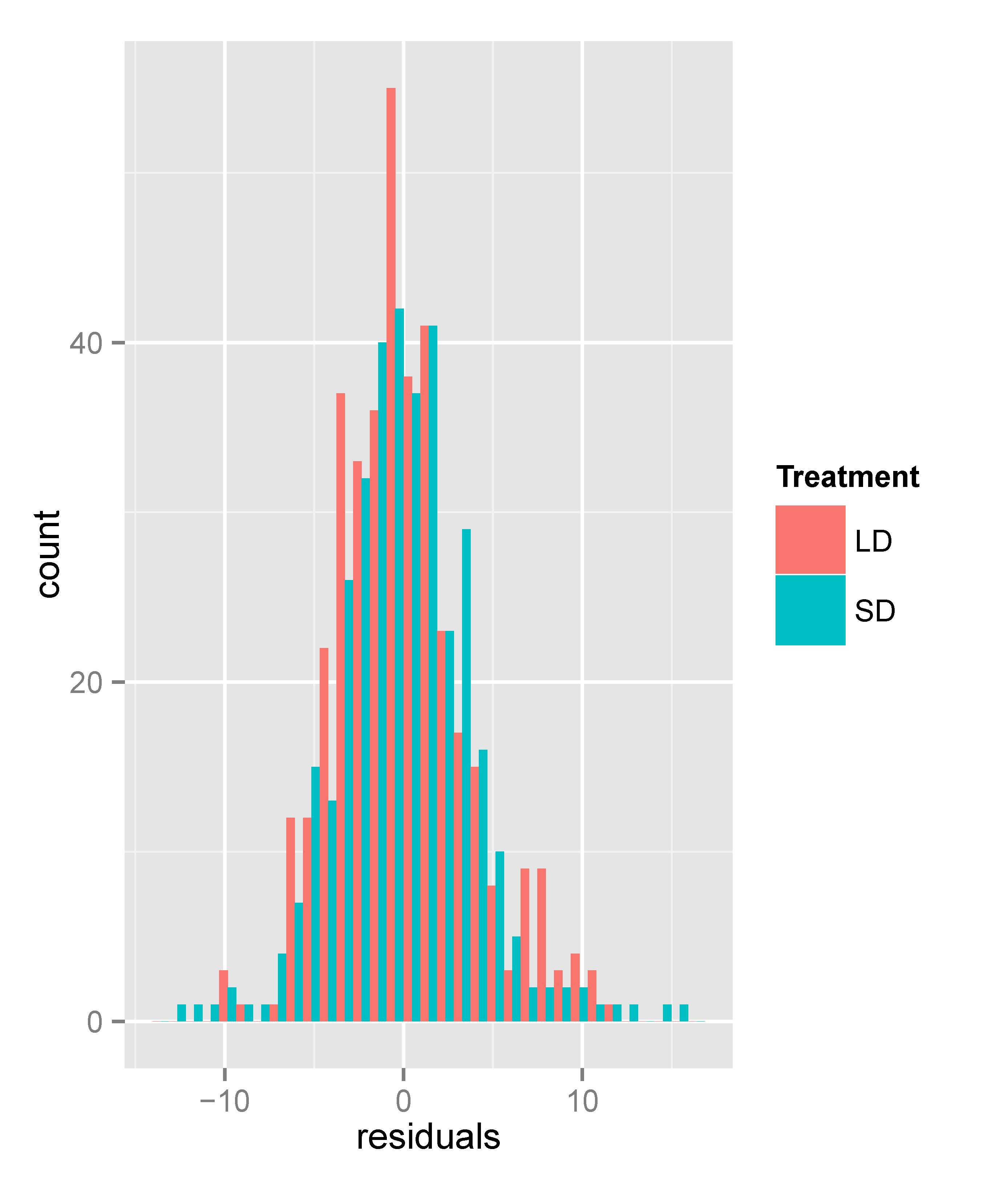

Supplement: Figure S3 — Histogram of residual errors for the number of leaf nodes at flowering (LNF). Plants from the LMDH population were grown under long day (LD) and short day (SD) conditions in a glasshouse-based experiment. (TIF) [file pone.0102611.s003.tif]

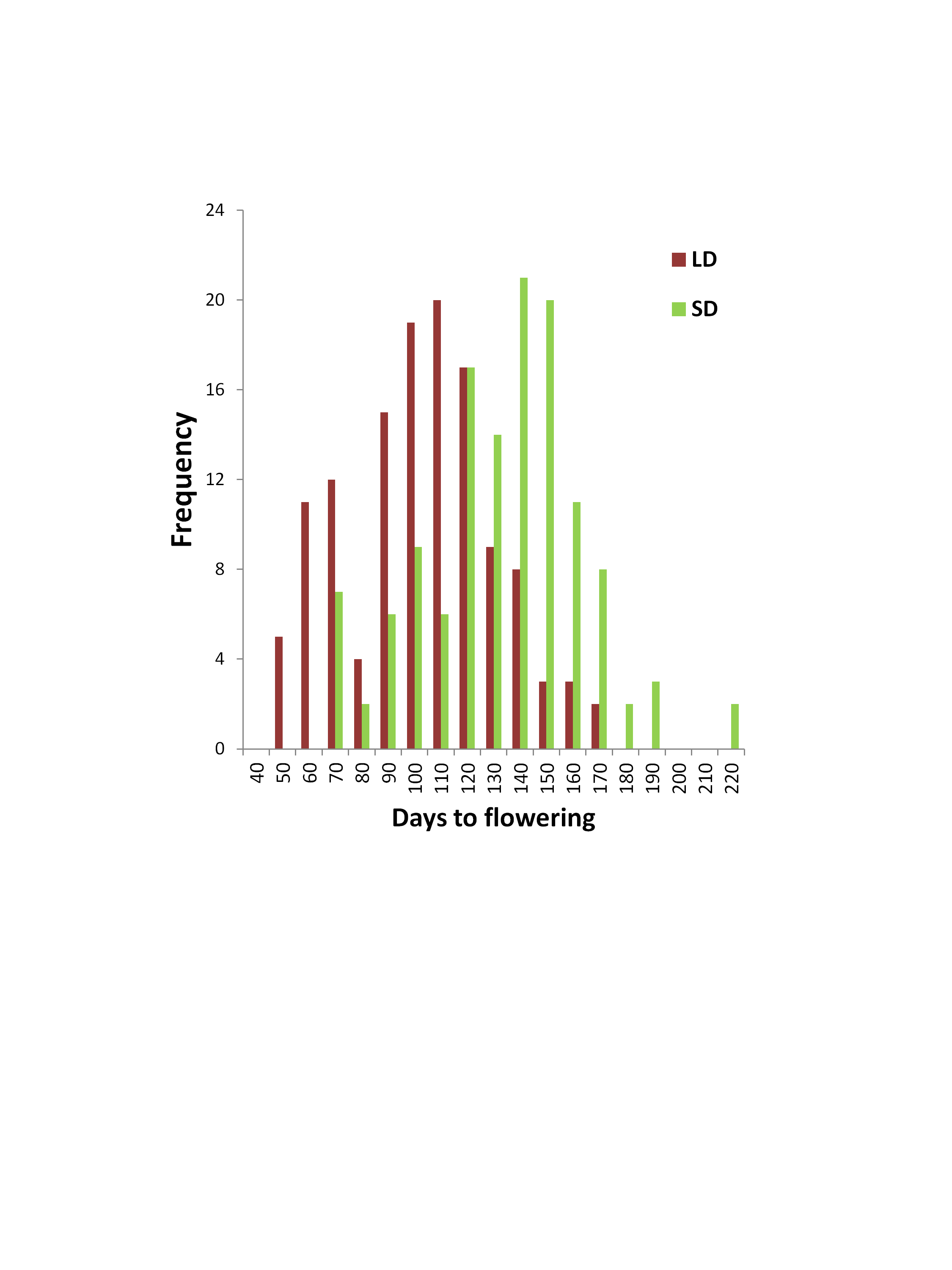

Supplement: Figure S4 — Frequency distribution for days to flowering (DTF) in the LMDH population. Plants were grown under long day (LD) and short day (SD) conditions in a glasshouse-based experiment. The mean DTF for Monty-028DH was 90.5 days (LD) and 104.4 days (SD). The mean DTF for Lynx-037DH was 110.6 days (LD) and 141.0 days (SD). (TIF) [file pone.0102611.s004.tif]

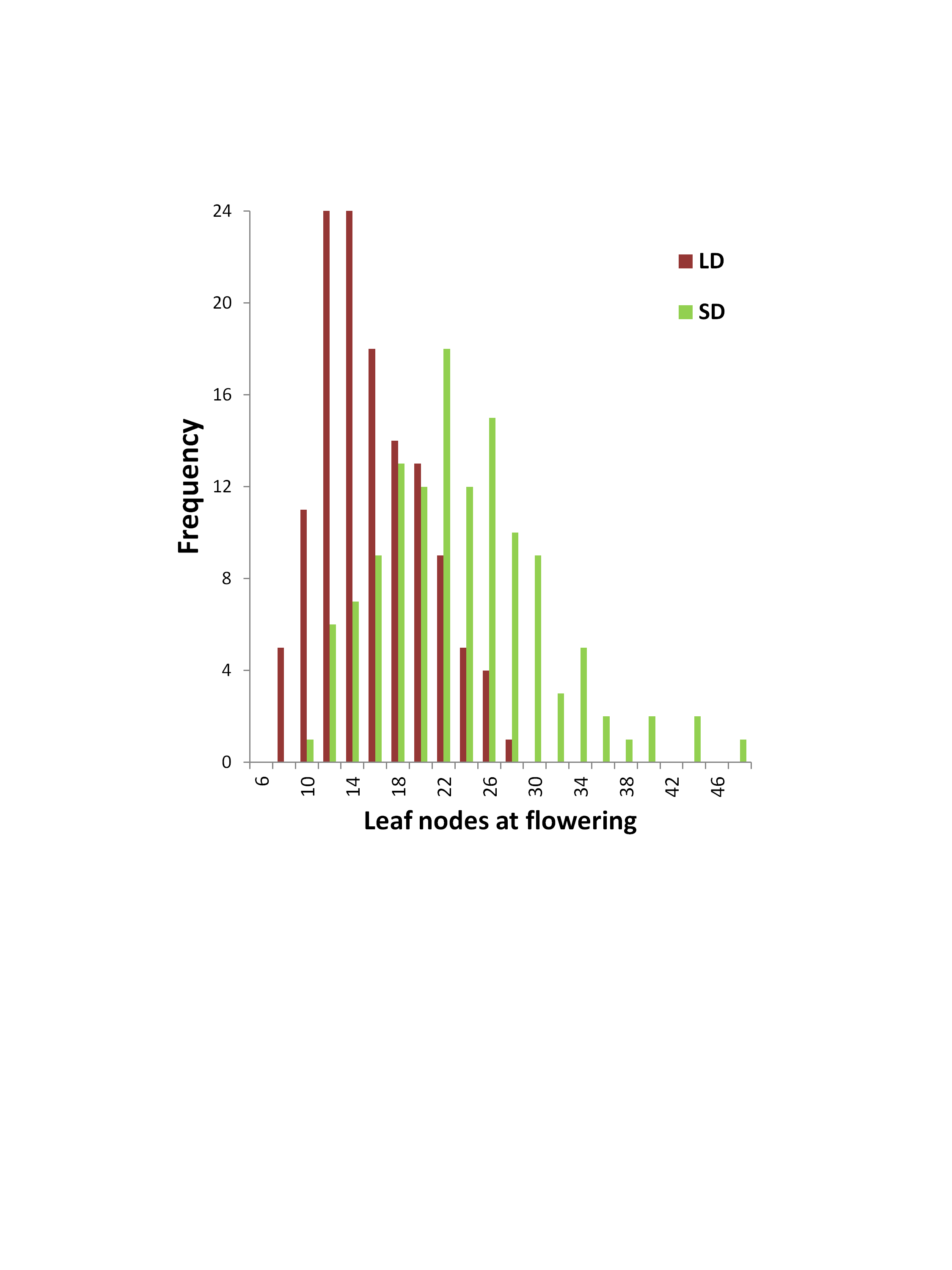

Supplement: Figure S5 — Frequency distribution for number of leaf nodes at flowering (LNF) in the LMDH population. Plants were grown under long day (LD) and short day (SD) conditions in a glasshouse-based experiment. The mean LNF for Monty-028DH was 15.3 (LD) and 18.8 (SD). The mean LNF for Lynx-037DH was 18.4 (LD) and 23.9 (SD). (TIF) [file pone.0102611.s005.tif]
