# Supplementary material for: Quantitative Trait Loci for Thermal Time to Flowering and Photoperiod Responsiveness Discovered in Summer Annual-Type Brassica napus L
Source: PLoS One. 2014 Jul 25;9(7):e102611. doi: 10.1371/journal.pone.0102611 (PMC4111298; doi:10.1371/journal.pone.0102611)
Supplement: Text S1 — Base-line marker model, M0, in asreml syntax. (DOCX) [file pone.0102611.s010.docx]

**Base-line marker model (M0)**

The ASReml-R [42] syntax for fitting the base-line marker model, M0, is as follows:

M0.asr <- asreml(LNF ~ Trt*Idtype, random = ~ US(Trt):giv(MID) + US(Trt):ide(MID) + Rep + Bench + Bench:Block, rcov =~ at(Bench):ar1(Column):ar1(Row), ginverse = list(MID = fullMp.dinv), data=all.df, na.method.X=’include’)

where

- all.df is the data-frame containing the y-variable of interest (here LNF) and all the factors defining the experiment, namely
  - Trt: a factor with 2 levels (“LD” and “SD”) indexing the daylength treatments,
  - Idtype: a factor with 15 levels, namely “DH” for all the doubled haploid lines and 14 other levels corresponding to the parents, reciprocal F1s, control lines and DH lines without marker data
  - MID: a factor with 128 levels indexing the DH lines with marker data. Note that lines with no marker data will have an MID value of “NA” (the missing value indicator)
  - Rep: a factor with 2 levels indexing the day-length treatment replicates (groups of 2 benches)
  - Bench: a factor with 4 levels indexing benches
  - Column: a factor with 10 levels indexing columns of pots within a bench
  - Row: a factor with 20 levels indexing rows of pots within a bench
  - Block: a factor with 2 levels indexing replicate blocks for lines that were replicated within a bench. On each bench, Block 1 = Columns 1-5 and Block2 = Columns 6-10
- The term Trt*Idtype is fitted in the fixed part of the model, and fits fixed main effects for treatments as well as fitting fixed Idtype effects for each treatment, thereby excluding the latter from the sources of variation associated with the genetic effects (see main text).
- The term US(Trt):giv(MID) is fitted in the random part of the model, and fits random MID effects (i.e. for the DH lines with marker data) for each treatment. The use of the “giv” variance function for MID allows the inclusion of marker information using the approach of Stranden and Garrick [40]. The “giv” function links to the object given in the “ginverse” argument and here this is the data-frame “fullMp.dinv”, the rows of which correspond to the non-zero elements of the lower triangle of the inverse of the matrix MM^T^, where M is the 128 x 329 matrix of marker data and M^T^ is its transpose. Thus the term US(Trt):giv(MID) fits random marker effects for each treatment. The unstructured (US) variance model for Trt means there is a separate marker variance for each treatment and a covariance between treatments.
- The term US(Trt):ide(MID) is fitted in the random part of the model, and fits random MID effects for each treatment. The use of the “ide” variance function for MID means that the term fits random polygenic effects for each treatment. The unstructured variance model for Trt means there is a separate polygenic variance for each treatment and a covariance between treatments.
- The terms Rep, Bench and Bench:Block are fitted in the random part of the model, so fit random effects to accommodate the blocking structure of the experimental design (see main text).
- The variance structure for the residuals (as supplied in the “rcov” formula) comprises a spatial correlation structure for each bench, namely an autoregressive process of order 1 (“ar1”) for both the row and column dimensions (see [41]).
